# Measuring competence in central venous catheterization: a systematic-review

**DOI:** 10.1186/2193-1801-3-33

**Published:** 2014-01-17

**Authors:** Irene WY Ma, Nishan Sharma, Mary E Brindle, Jeff Caird, Kevin McLaughlin

**Affiliations:** Department of Medicine, University of Calgary, Calgary, AB Canada; W21C, University of Calgary, 3330 Hospital Dr NW, T2N 4N1 Calgary, AB Canada; Department of Surgery, University of Calgary, Calgary, AB Canada

**Keywords:** *Clinical competence*, *Checklist*, *Catheterization*, *Central venous*, *Medical education*

## Abstract

**Objectives:**

Central venous catheterization is a complex procedural skill. This study evaluates existing published tools on this procedure and systematically summarizes key competencies for the assessment of this technical skill.

**Methods:**

Using a previously published meta-analysis search strategy, we conducted a systematic review of published assessment tools using the electronic databases PubMed, MEDLINE, Education Resource Information Center (ERIC), the Cumulative Index to Nursing and Allied Health Literature (CINAHL), Excerpta Medica, and Cochrane Central Register of Controlled Trials. Two independent investigators abstracted information on tool content and characteristics.

**Results:**

Twenty-five studies were identified assessing a total of 147 items. Tools used for assessment at the bedside (clinical tools) had a higher % of items representing “preparation” and “infection control” than tools used for assessment using simulation (67 ± 26% vs. 32 ± 26%; p = 0.003 for “preparation” and 60 ± 41% vs. 11 ± 17%; p = 0.002 for “infection control”, respectively). Simulation tools had a higher % of items on “procedural competence” than clinical tools (60 ± 36% vs. 17 ± 15%; p = 0.002). Items in the domains of “Team working” and “Communication and working with the patient” were frequently under-represented.

**Conclusion:**

This study presents a comprehensive review of existing checklist items for the assessment of central venous catheterization. Although many key competencies are currently assessed by existing published tools, some domains may be under-represented by select tools.

**Electronic supplementary material:**

The online version of this article (doi:10.1186/2193-1801-3-33) contains supplementary material, which is available to authorized users.

## Background

Central venous catheterization is a procedure that is commonly performed, with an estimated 15 million central-line-days per year in the intensive care units in U.S. hospitals (Mermel 2000). Because training using simulation has been previously shown to be associated with improved performance outcomes as well as clinical outcomes (Ma et al. 2011; Barsuk et al. 2009b), multiple institutions have implemented simulation-based training programs (Ma et al. 2011; Cook et al. 2011). These training programs require significant human and material resources (Ogden et al. 2007). Thus, to evaluate the return on such departmental investments, assessment tools that yield valid and reliable data are needed in order to evaluate procedural competence of those who underwent training (Evans and Dodge 2010).

For the assessment of technical skills, traditionally, there have been two general approaches: either using checklists or global rating scales; a combination of both approaches may also be considered (Lammers et al. 2008). A checklist consists of a list of observable behaviors organized in a consistent manner, which then allows the evaluator to record the presence or absence of the demonstrated behavior (Hales et al. 2008). Global rating scales, on the other hand, use a Likert scale for rating either an overall impression of the performance or on individual items within a performance (Bould et al. 2009).

Because steps in a procedure are often sequential and predictable, it is felt that checklists may be better suited for the assessment of technical skills, as they are felt to be more objective than global rating scales (Lammers et al. 2008; Evans et al. 2005). However, the pitfalls of using checklists have been extensively debated in the health professional education literature (Norman et al. 1991; Van Der Vleuten et al. 1991; Hodges et al. 1999; Swartz et al. 1999; Epstein and Hundert 2002). In the hands of expert raters, global rating scales may in fact demonstrate better psychometric properties than checklists (Hodges and McIlroy 2003; Regehr et al. 1998; Ma et al. 2012). Despite this, checklists continue to be commonly used in the assessment of procedural skills. For central venous catheterization, in 2009 alone, there were seven publications that included assessment tools, each of which used a checklist (Evans and Dodge 2010).

In the evaluation of any skill, a clear understanding of the underlying task is critical. Items in the assessment tool should be both relevant and representative of the task in question (American Educational Research Association, American Psychological Association, & National Council on Measurement in Education. Standards for educational and psychological testing. 1999). In a systematic review of checklists for procedural skills in general, seven themes were identified (McKinley et al. 2008). These include: 1) Procedural competence, 2) Preparation, 3) Safety, 4) Communication and working with the patient, 5) Infection control, 6) Post-procedural care, and 7) Team-working. In this review, a third to a half of the checklists did not assess for key competencies in the domains of “infection control” and “safety” (McKinley et al. 2008). Unfortunately, incompetence in these same domains has significant adverse clinical consequences. Therefore, it may be problematic to simply borrow an existing published tool and assume that it would evaluate procedural competency accurately.

The objective of this study is to review existing assessment tools for rating central venous catheterization and determine the individual steps and key competencies evaluated by these tools. This information can help 1) better define the underlying task of central venous catheterization itself, and 2) assist evaluators in deciding which tools to use. To accomplish the above objective, we conducted a systematic review of published evaluation tools used during direct observation of performances of central venous catheterization. We used the database of our recently published systematic review of simulation-based education on central venous catheterization (Ma et al. 2011) as the basis of this current study.

## Results

### Search results and article overview

**Figure 1:**
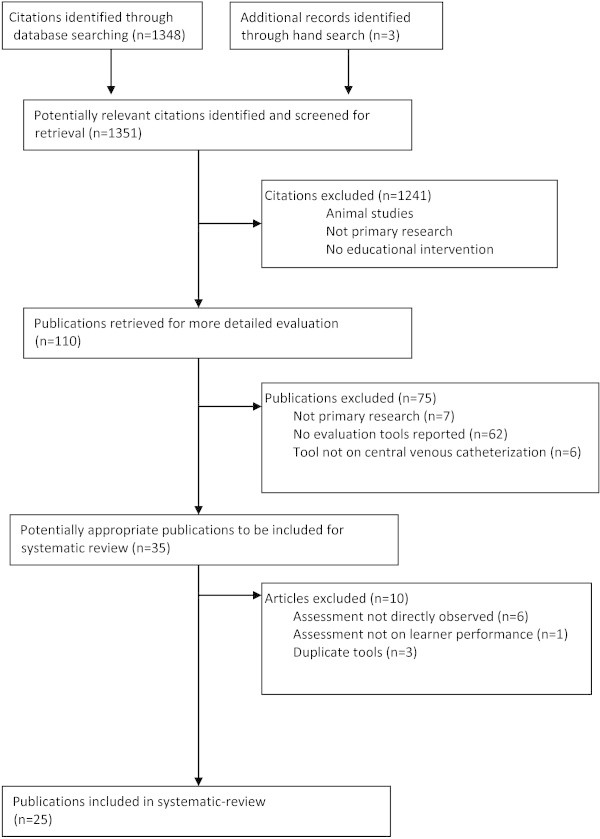
**Flow diagram of study selection process.**

Our previous search strategy from our systematic review (Ma et al. 2011) yielded 110 articles (Figure 1). These 110 articles resulted from excluding 1,241 articles from the initial search of 1,351 citations, (kappa 0.87; 95% CI 0.82-0.92).

In this review, from these 110 publications, 75 articles were excluded (Figure 1). Agreement for this stage was high (kappa 0.82; 95% CI 0.71-0.93). Thus, 35 articles were considered for review. Of the 35 articles, an additional 10 articles were excluded (kappa 0.85; 95% CI 0.66-1.00). A final pool of 25 publications was included in this systematic review. Figure 1 illustrates the results of the study selection process.

### Baseline description of tools

Overall, a total of 147 items were included in the assessment tools in 25 studies (Additional file 1). Median number of items included per study was 17 (IQR 8–22; range 2–63). All studies (100%) reported using checklists (using at least one binary item for assessing central venous catheterization skills). Only six studies reported also using global rating scales (Britt et al. 2009; Huang et al. 2009; Lee et al. 2009; Millington et al. 2009; Murphy et al. 2008; Ramakrishna et al. 2005). Other baseline characteristics of the tools are listed in Table 1.

**Table 1:** **Baseline characteristics of 25 studies describing directly observed central venous catheterization performances**

Study	Observers	Learner population	No. of learners observed	No. of procedures observed	Live vs video	Sites tested	Ultrasound used	Evaluations on patients (clinical) vs simulators	Checklist used (y/n); no of items	Global rating scale used (y/n); no. of items	Additional items assessed (y/n); no. of items
Barsuk et al. (2009c)	Faculty	MICU residents	28	N/A	Live and video	IJ, SC	Y	Simulators	Y; 27	N	N
Barsuk et al. (2009a)	Faculty	Nephrology fellows	18	N/A	Live and video	IJ	Y	Simulators	Y; 27	N	N
Berenholtz et al. (2004)	Nurses	ICU residents	N/A	64	Live	N/A	N/A	Clinical	Y; 8	N	N
Blaivas and Adhikari (2009)	Faculty	Emergency medicine residents	25	25	Live	IJ	Y	Simulators	Y; 2	N	Y; 1
Britt et al. (2009)	Trauma fellow or critical care surgeon	Junior surgery residents	34	73	Live	N/A	N/A	Clinical	Y; 14	Y; 2	Y; 1
Carvalho (2007)	Medical students	Medical students	9	N/A	Live	IJ, SC	Y	Simulators	Y; 1	N	Y; 2
Coopersmith et al. (2002)	Nurses	Residents in surgery, anesthesiology, emergency medicine, nurse practioner	N/A	16	Live	IJ, SC, Fem	N/A	Clinical	Y; 9	N	N
Costello et al. (2008)	N/A	N/A	N/A	N/A	Live	IJ, SC, Fem	N/A	Clinical	Y; 18	N	N
Dong et al. (2010)	Faculty and Fellow	Residents in anesthesiology, internal medicine, emergency medicine, general surgery, attending faculty	105	N/A	Video	IJ, SC	Y	Simulators	Y; 15	N	Y; 3
Evans et al. (2009)	Hired independent raters	PGY-1 and PGY-2 residents	N/A	N/A	Live and video	IJ, SC, Fem	Y	Simulators	Y; 63	N	Y; 2
Huang et al. (2009)	Faculty	Internal Medicine residents	42	94	Video	SC	N	Simulators	Y; 22	Y; 1	Y; 1
Kilbourne et al. (2009)	Study authors	Surgical or emergency medicine residents	N/A	86	Video	SC	N	Clinical	Y;6	N	Y; 2
Lee et al. (2009)	Expert reviewers	Emergency medicine residents	16	N/A	Video	IJ	Y	Simulators	Y; 19	Y;1	Y;2
Lobo et al. (2005)	Infection control staff	Medical residents	N/A	44	Live	IJ, SC, Fem	N/A	Clinical	Y;9	N	N
McKee et al. (2008)	Nurses	Pediatric anesthesiologists, surgeons, pediatric surgical staff, critical care medical staff	N/A	43	Live	N/A	N/A	Clinical	Y; 5	N	N
Millington et al. (2009)	Faculty	Medical residents	30	N/A	Video	IJ	N	Simulators	Y; 10	Y;5	Y;3
Murphy et al. (2008)	“Assessors”	Medical students	30	N/A	Video	IJ	N	Simulator	Y;20	Y;7	Y;1
Papadimos et al. (2008)	Independent observers	Residents in anesthesiology and surgery	N/A	85	Live	N/A	Available	Clinical	Y; 7	N	N
Ramakrishna et al. (2005)	Cardiologists	PGY2 medical residents	20	N/A	Live	IJ	Available	Clinical	Y;7	Y;1	N
Rosen et al. (2009)	Senior medical residents	Incoming medical residents	20	60	Live	IJ	Y	Chickens	Y; 22	N	N
Stone et al. (2010)	Faculty	Senior medical students and PGY-1 emergency medicine residents	39	N/A	Live	N/A	Y	Simulators	Y; 1	N	Y; 1
Velmahos et al. (2004)	Faculty	Surgical interns	26	N/A	Live	N	N	Clinical	Y;15	N	Y;3
Wall et al. (2005)	Nurses	“Trainees” in MICU	N/A	≥5	Live	IJ, SC, Fem	N/A	Clinical	Y;22	N	Y; 2
Xiao et al. (2007)	Faculty	Trauma residents	50	73	Video	IJ, SC, Fem	N/A	Clinical	Y;13	N	N
Yilmaz et al. (2007)	N/A	N/A	N/A	356	Live	N/A	N/A	Clinical	Y; 2	N	N

### Procedural checklists

Except for two studies, checklist items were scored in a binary fashion in general. One study (Ramakrishna et al. 2005) used a Likert scale of 1–5 (1=”very unsatisfactory”; 3=”neutral”; 5=”very satisfactory”) to score the seven items in the checklist, while the other study (Rosen et al. 2009) used a behaviorally anchored scale of 0–5 with a descriptor for each score to rate each of the 22 checklist items: (0=”displays complete unfamiliarity with the step, needs visual and verbal instruction in order to perform the step [‘stumped’], or omits step completely”; 5 = “executes procedure step independently, smoothly, with total confidence, and without error.”) The remaining studies scored checklist items in a binary fashion.

### Thematic content of checklist items

There were 11 checklists applied to assessments of procedural performances on simulators (simulation checklists) and 14 checklists applied to assessments of procedural performances on patients (clinical checklists) (Table 1).

Clinical checklists had a higher percentage of items representing “Preparation” and “Infection control” than simulation checklists (67 ± 26% vs. 32 ± 26%; p = 0.003 for “Preparation” and 60 ± 41% vs. 11 ± 17%; p = 0.002 for “Infection control”, respectively). Simulation checklists, on the other hand, had a higher percentage of items on “Procedural competence” than clinical checklists (60 ± 36% vs. 17 ± 15%; p = 0.002).

### Representation and underrepresentation of themes

A number of checklists were comprehensive in their representation of themes (Table 2). For example, six checklists (20%) contained at least one item in each of the seven domains (Barsuk et al. 2009a; Barsuk et al. 2009c; Evans et al. 2009; Huang et al. 2009; Wall et al. 2005; Dong et al. 2010). “Preparation” and “Infection control” were assessed in most checklists: only three checklists (12%) contained no items on “Preparation” (Blaivas and Adhikari 2009; Carvalho 2007; Stone et al. 2010) and only four checklists (16%) contained no items on “Infection control” (Blaivas and Adhikari 2009; Carvalho 2007; Kilbourne et al. 2009; Stone et al. 2010).

**Table 2:** **Themes represented by checklist items in 25 studies with checklists**

Study	Total no. of items	Preparation - no. items (%)	Infection control – no. items (%)	Communication and working with the patient – no. items (%)	Team working- no. items (%)	Safety – no. items (%)	Procedural competence – no. items (%)	Post-procedure – no. items (%)
Barsuk et al. (2009c)	27	11 (41)	6 (22)	1 (4)	1 (4)	4 (15)	13 (48)	5 (19)
Barsuk et al. (2009a)	27	10 (37)	6 (22)	1 (4)	1 (4)	4 (15)	13 (48)	6 (22)
Berenholtz et al. (2004)	8	6 (75)	8 (100)	0 (0)	1 (13)	0 (0)	1 (13)	1 (13)
Blaivas and Adhikari (2009)	3	0 (0)	0 (0)	0 (0)	0 (0)	3 (100)	3 (100)	0 (0)
Britt et al. (2009)	14	6 (43)	2 (14)	0 (0)	1 (7)	7 (50)	9 (64)	1 (7)
Carvalho (2007)	1	0 (0)	0 (0)	0 (0)	0 (0)	1 (100)	1 (100)	0 (0)
Coopersmith et al. (2002)	9	6 (67)	7 (78)	0 (0)	0 (0)	1 (11)	0 (0)	3 (33)
Costello et al. (2008)	18	15 (83)	13 (72)	1 (6)	9 (50)	5 (28)	0 (0)	2 (11)
Dong et al. (2010)	15	11 (73)	6 (40)	2 (13)	1 (7)	3 (20)	3 (20)	1 (7)
Evans et al. (2005)	61	31 (51)	9 (15)	1 (2)	3 (5)	18 (30)	24 (39)	8 (13)
Huang et al. (2009)	22	12 (55)	5 (23)	1 (5)	1 (5)	2 (9)	11 (50)	1 (5)
Kilbourne et al. (2009)	6	1 (17)	0 (0)	0 (0)	0 (0)	2 (33)	5 (83)	0 (0)
Lee et al. (2009)	19	9 (47)	6 (32)	1 (5)	0 (0)	4 (21)	9 (47)	1 (5)
Lobo et al. (2005)	9	8 (89)	9 (100)	0 (0)	0 (0)	1 (11)	0 (0)	1 (11)
McKee et al. (2008)	5	4 (80)	5 (100)	0 (0)	1 (20)	0 (0)	0 (0)	1 (20)
Millington et al. (2009)	10	2 (20)	1 (10)	0 (0)	0 (0)	0 (0)	6 (60)	2 (20)
Murphy et al. (2008)	20	4 (20)	1 (5)	1 (5)	0 (0)	6 (30)	13 (65)	3 (15)
Papadimos et al. (2008)	7	6 (86)	7 (100)	0 (0)	1 (14)	0 (0)	1 (14)	2 (29)
Ramakrishna et al. (2005)	7	3 (43)	1 (14)	1 (14)	0 (0)	0 (0)	4 (57)	0 (0)
Rosen et al. (2009)	22	13 (59)	7 (32)	1 (5)	0 (0)	3 (14)	6 (27)	3 (14)
Stone et al. (2010)	1	0 (0)	0 (0)	0 (0)	0 (0)	1 (100)	1 (100)	0 (0)
Velmahos et al. (2004)	15	5 (33)	2 (13)	0 (0)	1 (7)	2 (13)	7 (47)	3 (20)
Wall et al. (2005)	22	17 (77)	9 (41)	2 (9)	2 (9)	4 (18)	2 (9)	3 (14)
Xiao et al. (2007)	13	13 (100)	12 (100)	0 (0)	0 (0)	0 (0)	0 (0)	0 (0)
Yilmaz et al. (2007)	2	2 (100)	2 (92)	0 (0)	0 (0)	0 (0)	0 (0)	0 (0)

Other themes were less well-represented by checklists: 13 checklists (52%) contained no items on “Team working”(Lee et al. 2009; Lobo et al. 2005; Millington et al. 2009; Murphy et al. 2008; Rosen et al. 2009; Ramakrishna et al. 2005; Blaivas and Adhikari 2009; Carvalho 2007; Stone et al. 2010; Kilbourne et al. 2009; Coopersmith et al. 2002; Xiao et al. 2007; Yilmaz et al. 2007); 14 checklists (56%) contained no items on “Communication and working with the patient” (Berenholtz et al. 2004; Blaivas and Adhikari 2009; Britt et al. 2009; Carvalho 2007; Coopersmith et al. 2002; Kilbourne et al. 2009; Lobo et al. 2005; McKee et al. 2008; Millington et al. 2009; Papadimos et al. 2008; Stone et al. 2010; Velmahos et al. 2004; Xiao et al. 2007; Yilmaz et al. 2007); seven checklists (28%) contained no items on “Post-procedure” (Ramakrishna et al. 2005; Blaivas and Adhikari 2009; Carvalho 2007; Stone et al. 2010; Kilbourne et al. 2009; Xiao et al. 2007; Yilmaz et al. 2007); seven checklists (28%) contained no items on “Safety” (Berenholtz et al. 2004; McKee et al. 2008; Millington et al. 2009; Papadimos et al. 2008; Ramakrishna et al. 2005; Xiao et al. 2007; Yilmaz et al. 2007); and six checklists (24%) contained no items on “Procedural competence” (Coopersmith et al. 2002; Costello et al. 2008; Lobo et al. 2005; McKee et al. 2008; Xiao et al. 2007; Yilmaz et al. 2007).

### Global rating scales and additional items assessed

Only six studies reported the use of global rating scales (Britt et al. 2009; Huang et al. 2009; Lee et al. 2009; Millington et al. 2009; Murphy et al. 2008; Ramakrishna et al. 2005), all of which were used in conjunction with checklist items (Table 3). The median number of items assessed was 2 (IQR 1–5; range 1–7). Additional items assessed frequently included number of attempts and time taken to perform the procedure (Table 4).

**Table 3:** **Global rating scale assessed**

Study	No. of global rating scale items	Items	Scale used
Britt et al. (2009)	2	Resident comfort; Resident ability	1-5
Huang et al. (2009)	1	Overall performance	Anchored 1–5
			(1 = “unable to complete procedure without assistance”, 3 = “ demonstrates essential skills to complete procedure”, 5=”demonstrates mastery of procedure skills”)
Lee et al. (2009)	1	Overall performance	1-7 (Poor to excellent)
Millington et al. (2009)	5	Time and motion; Instrument handling; Flow of operation and forward planning; Knowledge of instruments;	1-5 behaviorally anchored scales^1^
		Overall rating	1–5 (1=”overall does not meet expectations”), 5 =”superior, exceeds expectations”)
Murphy et al. (2008)	7	Respect for tissue; Time and motion; Instrument handling; Knowledge of instruments; Use of assistants; Flow of procedure and forward planning; Knowledge of specific procedure	1-5 behaviorally anchored scales^1^
Ramakrishna et al. (2005)	1	Overall perception: Resident is capable of *independently* performing central line procedures	1-5 (1 = “Strongly disagree”, 3 = “Neutral”, 5 = “Strongly agree”)

^1^Global rating scale based on scale from Reznick R, Regehr G, MacRae H et al. Am J Surg 1997; 173(3):226–30.

**Table 4:** **Additional items assessed**

Study	No. of additional items assessed	Items
Blaivas and Adhikari (2009)	1	No. of times posterior wall penetrated
Britt et al. (2009)	1	Average sticks to cannulation
Carvalho (2007)	2	No. of attempts required to cannulate the vessel;
		Time from skin penetration to successful guidewire insertion and needle removal
Dong et al. (2010)	3	Number of venipuncture attempts;
		Number of skin entries;
		Procedural time (from initial greeting of the ‘patient’ until successful catheterization)
Evans and Dodge (2010)	2	Total number of attempts to cannulate vein with large bore needle;
		Time to completion
Huang et al. (2009)	1	Number of passes
Kilbourne et al. (2009)	2	Number of insertion attempts;
		Number of unsuccessful failure
Lee et al. (2009)	2	Number of attempts;
		Time needle touches skin and time vessel successfully puncture
Millington et al. (2009)	3	Number of attempts to locate the vein;
		Number of attempts to insert the catheter;
		Total time for procedure
Murphy et al. (2008)	1	Time taken to complete the procedure
Stone et al. (2010)	1	Time from first synthetic skin puncture until “flash”;1
Velmahos et al. (2004)	3	Number of attempts to locate the vein;
		Number of attempts to insert the catheter;
		Time to complete procedure.
Wall et al. (2005)	2	List all sites where insertion was attempted;
		How many different needle sticks did the patient receive (number of skin breaks)?

### Validity and reliability evidence for the assessment tools

Inter-rater reliability was reported for 12 (48%) of the studies (Barsuk et al. 2009a; Barsuk et al. 2009c; Dong et al. 2010; Evans et al. 2009; Huang et al. 2009; Lee et al. 2009; Millington et al. 2009; Murphy et al. 2008; Rosen et al. 2009; Kilbourne et al. 2009; Stone et al. 2010; Xiao et al. 2007), reporting a range of reliability coefficients and absolute agreement [range 0.43 (Millington et al. 2009) to 0.97(Evans et al. 2009)]. Only 12 studies (48%) specified the process used for content validation (Velmahos et al. 2004; Barsuk et al. 2009a; Barsuk et al. 2009c; Costello et al. 2008; Dong et al. 2010; Evans et al. 2009; Huang et al. 2009; Lee et al. 2009; Rosen et al. 2009; Wall et al. 2005; Kilbourne et al. 2009; Coopersmith et al. 2002).

## Discussion

Our study identified 25 published tools for the assessment of procedural skills in central venous catheterization. All of these tools used at least one item that is scored in a binary checklist fashion and only six studies reported using a global rating scale.

Our study identified that only 20% of the assessment tools incorporated at least one item in each of the seven key procedural competence domains; the majority of tools did not assess for competency in the domains of “Team working” and “Communication and working with the patient.”

In an effort to improve clinical outcomes through the use of simulation-based training, trainers need to be mindful of assessing domains that have implications on patient safety, such as “Team working”, “Safety” and “Infection control.” Therefore, the tool, wherever possible, should strive to aim for including items in as many of the seven key competency domains as possible. Failing the ability to assess the procedure in a systematic and comprehensive manner, consideration should be made towards using a global rating scale instead.

Not every tool is created equally. Tools are frequently created with specific purposes in mind. Thus for an evaluator wishing to borrow a pre-existing assessment tool from the published literature for the purposes of assessments, this study provides a comprehensive list of assessment items to facilitate educators and assessors in choosing an appropriate tool.

There are some limitations in this systematic review that impact on the interpretation of our study’s conclusions. First, despite our systematic review including only publications that included an educational intervention, the assessment purposes of the studies were not uniform. Tools designed to be used by nurses for the purposes of documenting infectious risks only or tools designed for the purposes of assessing performances on simulators are unlikely to be as comprehensive as tools designed to assess for overall competence of procedural skills on patients. Indeed, our results suggest that clinical checklists were more focused on steps involving preparation and infection control than simulation checklists, while simulation checklists were more focused on procedural technical competence itself. Therefore, the contextual features of each published tool are important to recognize, since ultimately, validity of any assessment tool refers to the “degree to which evidence and theory support the interpretations of test scores entailed by the proposed uses of tests” (American Educational Research Association, American Psychological Association, & National Council on Measurement in Education. Standards for educational and psychological testing. 1999). Second, despite contacting authors to obtain the actual checklists, although a number did provide these (Wall et al. 2005; Lobo et al. 2005; Costello et al. 2008), a few studies were excluded because of a lack of response from the authors.

Despite these limitations, this study has a number of strengths. By providing a systematic and comprehensive evaluation and description of existing tools on central venous catheterization, this study facilitates educators, researchers, or hospital administrators wishing to use, study or develop assessments tools on assessing for competency in this procedure. Furthermore, this study compiles, for the first time, a “catalog” of all the potential aspects of the procedure that could be assessed (see Additional file 1). This “catalog” represents the end product of work from multiple groups using various methods such as cognitive task analysis, literature review, and expert panels.

## Conclusions

In conclusion, in this systematic review of published assessment tools on central venous catheterization, we present a comprehensive list of assessment items. We found that the use of procedural checklists far outnumber the use of global rating scales. The majority of these tools did not assess for competency in the domains of “Team working” and “Communication and working with the patient.” Lastly, the rigor in which the tools were developed greatly varied.

## Methods

### Data sources and search strategy

The search strategy was previously published (Ma et al. 2011). In short, searches for relevant articles published between January 1950 and May 2010 were conducted on the following databases: PubMed, MEDLINE, Education Resource Information Center (ERIC), the Cumulative Index to Nursing and Allied Health Literature (CINAHL), Excerpta Medica, and Cochrane Central Register of Controlled Trials. Our search strategy was developed with the assistance of a research librarian and used the following keywords: *catheterization, central venous; catheterization; catheter$; jugular veins; subclavian veins;* and *femoral veins.* These terms were searched as subject headings, medical subject heading, and text words, and combined with the Boolean operator “and” with education terms. Education terms used were: *education; learning; teaching;* and *teach$*. We did not place a language restriction on the search. The initial screening of search results was done independently by two authors (I.M., M.B.), using titles and abstracts. Additional hand search for references in included articles and relevant review articles was conducted. From this initial search (Ma et al. 2011), citations that were clearly not primary research, involved animal studies, or did not involve an educational intervention were excluded. For the remaining citations, full-length articles were retrieved.

### Selection of articles

From these full-length articles, we included primary research articles that described the assessments of central venous catheterization skills under direct observation. That is, we excluded articles where the procedures were performed without anyone observing the procedures. We also excluded studies on peripherally-placed venous access devices as well as studies without an educational intervention. Articles that did not provide an assessment tool or articles that did not include descriptions of assessment items were excluded. For studies where only descriptions of assessment items were reported without provision of the assessment tool, we contacted the authors to obtain the full tool. Selection of articles was done independently by two authors (I.M., N.S.), with disagreements resolved by consensus.

### Data extraction

Independent data abstraction on baseline characteristics of each study was performed by two authors (IM, NS) using a standardized data form. Information on learner population, observers, and tools was obtained from each publication. We also abstracted information on whether or not the tool was used on patients (clinical) or on simulators.

We defined any item scored an observable action item in a binary fashion (y/n) as being part of a “checklist,” whether or not the authors specified the use of the tool as a “checklist.” For example, if “need for help from senior resident”(Velmahos et al. 2004) is routinely assessed in the observed performances, this item is considered to be one of the checklist items. Checklist items scored in a non-binary fashion are also included. We defined global rating scale items as those that use a Likert scale for rating either an overall impression of the performance or on individual qualities within the performance (Bould et al. 2009).

### Classification of items into seven competency themes

Each checklist item was classified by two authors (IM, MB) according to one or more of the seven competency themes previously identified (McKinley et al. 2008): 1) Preparation, 2) Infection control, 3) Communication and working with the patient, 4) Team working, 5) Safety, 6) Procedural competence, and 7) Post-procedure.

Disagreements were resolved by consensus. Items may be classified into more than one theme. For example, an item on obtaining informed consent was classified into both “Preparation” as it involves assessing for indications and contraindications for the procedure (McKinley et al. 2008) as well as “Communication and working with the patient,” which involves sharing information about the procedure with the patient (McKinley et al. 2008).

We defined “Preparation” as any steps prior to the breach in patient skin (i.e. administration of anesthetics or insertion of needle). Steps after the administration of anesthetics but before securing of the catheters were considered part of “Procedural competence.” Lastly, we defined any steps including or after securing the catheter as “Post-procedure,” such as placement of dressing, obtaining chest x-rays, documentation of procedure, and equipment clean-up.

Immediate complications are included as assessment items only if they are part of the directly-observed evaluation. For example, carotid puncture, pneumothorax, hemothorax, malignant arrhythmia, and number of needle passes. Long-term complications such as catheter-related infections are excluded, as these “distal” outcomes may or may not be directly related to the learner performance.

### Statistical analysis

Data were analyzed using standard parametric and non-parametric methods. Comparisons of continuous variables between groups were performed using Student’s *t*-tests. Inter-rater agreement in study selection is estimated by the kappa statistic. All analyses were performed using SAS version 9.2 (SAS Institute Inc., Cary, NC, USA) and Stata 11.0 (StataCorp LP, College Station, TX).

## Electronic supplementary material

Additional file 1: **Checklist items for the 25 studies.** (DOC 345 KB)

## Abbreviations

95% CI: 95% confidence interval
IQR: Interquartile range.

## References

[CR1] (1999). Standards for educational and psychological testing.

[CR2] Barsuk JH, Ahya SN, Cohen ER, McGaghie WC, Wayne DB (2009). Mastery learning of temporary hemodialysis catheter insertion by nephrology fellows using simulation technology and deliberate practice. Am J Kidney Dis.

[CR3] Barsuk JH, Cohen ER, Feinglass J, McGaghie WC, Wayne DB (2009). Use of simulation-based education to reduce catheter-related bloodstream infections. Arch Intern Med.

[CR4] Barsuk JH, McGaghie WC, Cohen ER, Balachandran JS, Wayne DB (2009). Use of simulation-based mastery learning to improve the quality of central venous catheter placement in a medical intensive care unit. J Hosp Med.

[CR5] Berenholtz SM, Pronovost PJ, Lipsett PA, Hobson D, Earsing K, Farley JE, Milanovich S, Garrett-Mayer E, Winters BD, Rubin HR, Dorman T, Perl TM (2004). Eliminating catheter-related bloodstream infections in the intensive care unit. Crit Care Med.

[CR6] Blaivas M, Adhikari S (2009). An unseen danger: Frequency of posterior vessel wall penetration by needles during attempts to place internal jugular vein central catheters using ultrasound guidance. Crit Care Med.

[CR7] Bould MD, Crabtree NA, Naik VN (2009). Assessment of procedural skills in anaesthesia. Br J Anaesth.

[CR8] Britt RC, Novosel TJ, Britt LD, Sullivan M (2009). The impact of central line simulation before the ICU experience. Am J Surg.

[CR9] Carvalho P (2007). Early introduction to central line placement: a curriculum for medical students. Med Educ.

[CR10] Cook DA, Hatala R, Brydges R, Zendejas B, Szostek JH, Wang AT, Erwin PJ, Hamstra SJ (2011). Technology-enhanced simulation for health professions education: a systematic review and meta-analysis. JAMA.

[CR11] Coopersmith CM, Rebmann TL, Zack JE, Ward MR, Corcoran RM, Schallom ME, Sona CS, Buchman TG, Boyle WA, Polish LB, Fraser VJ (2002). Effect of an education program on decreasing catheter-related bloodstream infections in the surgical intensive care unit. Crit Care Med.

[CR12] Costello JM, Morrow DF, Graham DA, Potter-Bynoe G, Sandora TJ, Laussen PC (2008). Systematic intervention to reduce central line–associated bloodstream infection rates in a pediatric cardiac intensive care unit. Pediatrics.

[CR13] Dong Y, Suri HS, Cook DA, Kashani KB, Mullon JJ, Enders FT, Rubin O, Ziv A, Dunn WF (2010). Simulation-based objective assessment discerns clinical proficiency in central line placement: a construct validation. Chest.

[CR14] Epstein RM, Hundert EM (2002). Defining and assessing professional competence. JAMA.

[CR15] Evans LV, Dodge KL (2010). Simulation and patient safety: evaluative checklists for central venous catheter insertion. Qual Saf Health Care.

[CR16] Evans AW, McKenna C, Oliver M (2005). Trainees’ perspectives on the assessment and self‒assessment of surgical skills. Assess Eval High Educ.

[CR17] Evans LV, Morse JL, Hamann CJ, Osborne M, Lin Z, D’Onofrio G (2009). The development of an independent rater system to assess residents’ competence in invasive procedures. Acad Med.

[CR18] Hales B, Terblanche M, Fowler R, Sibbald W (2008). Development of medical checklists for improved quality of patient care. International J Qual Health Care.

[CR19] Hodges B, McIlroy JH (2003). Analytic global OSCE ratings are sensitive to level of training. Med Educ.

[CR20] Hodges B, Regehr G, McNaughton N, Tiberius R, Hanson M (1999). OSCE checklists do not capture increasing levels of expertise. Acad Med.

[CR21] Huang GC, Newman LR, Schwartzstein RM, Clardy PF, Feller-Kopman D, Irish JT, Smith CC (2009). Procedural competence in internal medicine residents: validity of a central venous catheter insertion assessment instrument. Acad Med.

[CR22] Kilbourne MJ, Bochicchio GV, Scalea T, Xiao Y (2009). Avoiding common technical errors in subclavian central venous catheter placement. J Am Coll Surg.

[CR23] Lammers RL, Davenport M, Korley F, Griswold-Theodorson S, Fitch MT, Narang AT, Evans LV, Gross A, Rodriguez E, Dodge KL, Hamann CJ, Robey Iii WC (2008). Teaching and Assessing Procedural Skills Using Simulation: Metrics and Methodology. Acad Emerg Med.

[CR24] Lee AC, Thompson C, Frank J, Beecker J, Yeung M, Woo MY, Cardinal P (2009). Effectiveness of a novel training program for emergency medicine residents in ultrasound-guided insertion of central venous catheters. CJEM.

[CR25] Lobo RD, Levin AS, Brasileiro Gomes LM, Cursino R, Park M, Figueiredo VB, Taniguchi L, Polido CG, Costa SF (2005). Impact of an educational program and policy changes on decreasing catheter-associated bloodstream infections in a medical intensive care unit in Brazil. Am J Infect Control.

[CR26] Ma IW, Brindle M, Ronksley P, Lorenzetti D, Sauve R, Ghali W (2011). Use of simulation-based education to improve outcomes of central venous catheterization: a systematic review and meta-analysis. Acad Med.

[CR27] Ma IW, Zalunardo N, Pachev G, Beran T, Brown M, Hatala R, McLaughlin K (2012). Comparing the use of global rating scale with checklists for the assessment of central venous catheterization skills using simulation. Adv Health Sci Educ Theory Pract.

[CR28] McKee C, Berkowitz I, Cosgrove SE, Bradley K, Beers C, Perl TM, Winner L, Pronovost PJ, Miller MR (2008). Reduction of catheter-associated bloodstream infections in pediatric patients: Experimentation and reality. Pediatr Crit Care Med.

[CR29] McKinley RK, Strand J, Ward L, Gray T, Alun-Jones T, Miller H (2008). Checklists for assessment and certification of clinical procedural skills omit essential competencies: a systematic review. Med Educ.

[CR30] Mermel LA (2000). Prevention of intravascular catheter–related infections. Ann Intern Med.

[CR31] Millington SJ, Wong RY, Kassen BO, Roberts JM, Ma IW (2009). Improving internal medicine residents’ performance, knowledge, and confidence in central venous catheterization using simulators. J Hosp Med.

[CR32] Murphy MA, Neequaye S, Kreckler S, Hands LJ (2008). Should we train the trainers? Results of a randomized trial. J Am Coll Surg.

[CR33] Norman GR, Van Der Vleuten CPM, De Graaff E (1991). Pitfalls in the pursuit of objectivity: issues of validity, efficiency and acceptability. Med Educ.

[CR34] Ogden PE, Cobbs LS, Howell MR, Sibbitt SJ, DiPette DJ (2007). Clinical simulation: importance to the internal medicine educational mission. Am J Med.

[CR35] Papadimos T, Hensely S, Duggan J, Hofmann J, Khuder S, Borst M, Fath J (2008). Intensivist supervision of resident-placed central venous catheters decreases the incidence of catheter-related blood stream infections. Patient Saf Surg.

[CR36] Ramakrishna G, Higano ST, McDonald FS, Schultz HJ (2005). A curricular initiative for internal medicine residents to enhance proficiency in internal jugular central venous line placement. Mayo Clin Proc.

[CR37] Regehr G, MacRae H, Reznick RK, Szalay D (1998). Comparing the psychometric properties of checklists and global rating scales for assessing performance on an OSCE-format examination. Acad Med.

[CR38] Rosen BT, Uddin PQ, Harrington AR, Ault BW, Ault MJ (2009). Does personalized vascular access training on a nonhuman tissue model allow for learning and retention of central line placement skills? Phase II of the procedural patient safety initiative (PPSI-II). J Hosp Med.

[CR39] Stone MB, Moon C, Sutijono D, Blaivas M (2010). Needle tip visualization during ultrasound-guided vascular access: short-axis vs long-axis approach. Am J Emerg Med.

[CR40] Swartz MH, Colliver JA, Bardes CL, Charon R, Fried ED, Moroff S (1999). Global ratings of videotaped performance versus global ratings of actions recorded on checklists: a criterion for performance assessment with standardized patients. Acad Med.

[CR41] Van Der Vleuten CPM, Norman GR, De Graaff E (1991). Pitfalls in the pursuit of objectivity: issues of reliability. Med Educ.

[CR42] Velmahos GC, Toutouzas KG, Sillin LF, Chan L, Clark RE, Theodorou D, Maupin F (2004). Cognitive task analysis for teaching technical skills in an inanimate surgical skills laboratory. Am J Surg.

[CR43] Wall RJ, Ely EW, Elasy TA, Dittus RS, Foss J, Wilkerson KS, Speroff T (2005). Using real time process measurements to reduce catheter related bloodstream infections in the intensive care unit. Qual Saf Health Care.

[CR44] Xiao Y, Seagull FJ, Bochicchio GV, Guzzo JL, Dutton RP, Sisley A, Joshi M, Standiford HC, Hebden JN, Mackenzie CF, Scalea TM (2007). Video-based training increases sterile-technique compliance during central venous catheter insertion. Crit Care Med.

[CR45] Yilmaz G, Caylan R, Aydin K, Topbas M, Koksal I (2007). Effect of education on the rate of and the understanding of risk factors for intravascular catheter-related infections. Infect Control Hosp Epidemiol.

