# Supplementary material for: Measuring competence in central venous catheterization: a systematic-review
Source: Springerplus. 2014 Jan 17;3:33. doi: 10.1186/2193-1801-3-33 (PMC3909608; doi:10.1186/2193-1801-3-33)
Supplement: Supplementary file 1 — Additional file 1: Checklist items for the 25 studies. (DOC 345 KB) [file 40064_2013_791_MOESM1_ESM.doc]

| **Item** | **Description** | **Evans et al (2009)** | **Barsuk et al (2009b)** | **Barsuk et al (2009a)** | **Berenholtz et al (2004)** | **Blaivas and Adhikari (2009)** | **Britt et al (2009)** | **Carvalho (2007)** | **Coopersmith et al (2002)** | **Costello et al (2008)** | **Dong et al (2010)** | **Huang et al (2009)** | **Kilbourne et al (2009)** | **Lee et al (2009)** | **Lobo et al (2005)** | **McKee et al (2008)** | **Millington et al (2009)** | **Papadimos et al (2008)** | **Ramakrishna et al (2005)** | **Stone et al (2010)** | **Wall et al (2005)** | **Yilmaz et al (2007)** | **Murphy et al (2008)** | **Rosen et al (2009)** | **Velmahos et al (2004)** | **Xiao et al (2007)** |
| --- | --- | --- | --- | --- | --- | --- | --- | --- | --- | --- | --- | --- | --- | --- | --- | --- | --- | --- | --- | --- | --- | --- | --- | --- | --- | --- |
| 1 | Patient interaction |  |  |  |  |  |  |  |  |  |  |  |  |  |  |  |  |  | 1 |  | 2 |  |  |  |  |  |
| 2 | Verification of patient id |  |  |  |  |  |  |  |  |  | 1 |  |  |  |  |  |  |  |  |  |  |  |  | 0.5 |  |  |
| 3 | Obtains consent | 1 | 1 | 1 |  |  |  |  |  |  | 1 |  |  |  |  |  |  |  |  |  | 1 |  | 0.33 | 0.5 |  |  |
| 4 | Warns patient cold |  |  |  |  |  |  |  |  |  |  |  |  | 1 |  |  |  |  |  |  |  |  |  |  |  |  |
| 5 | Places patient on monitor | 1 |  |  |  |  |  |  |  |  |  |  |  |  |  |  |  |  |  |  |  |  |  |  |  |  |
| 6 | Positions patient (neck at 45 deg angle) | 1 |  |  |  |  |  |  |  |  |  |  |  |  |  |  |  |  |  |  |  |  |  |  |  |  |
| 7 | Trendelenberg position | 1 | 1 | 1 |  |  | 1 |  |  |  | 1 | 1 |  |  |  |  |  |  |  |  |  |  |  |  | 1 |  |
| 8 | Prepares entire neck area with chlorhexidine, cleans skin | 1 | 1 | 1 | 1 |  |  |  |  |  |  | 0.33 |  | 1 | 1 | 1 |  | 1 | 0.5 |  |  |  |  | 0.5 |  | 1 |
| 9 | Type of prep observed and recorded |  |  |  |  |  |  |  |  | 3 | 1 |  |  |  | 1.5 |  |  |  |  |  | 1 |  |  |  |  | 1 |
| 10 | Scrub time > 30 sec (or 2 min for groin line) |  |  |  |  |  |  |  |  | 2 |  | 0.33 |  |  |  |  |  |  |  |  |  |  |  |  |  |  |
| 11 | Dry time >30s |  |  |  |  |  |  |  |  | 1 |  | 0.33 |  |  |  |  |  |  |  |  |  |  |  |  |  | 1 |
| 12 | Repeat chlorhexidine |  |  |  |  |  |  |  |  |  |  |  |  | 1 |  |  |  |  |  |  |  |  |  | 0.5 |  |  |
| 13 | Uses sterile towel |  |  |  |  |  |  |  |  |  |  |  |  | 1 |  |  |  |  |  |  | 1 |  |  |  |  |  |
| 14 | Applies full drape (notation toward head) |  | 1 | 1 | 1 |  |  |  | 1 | 1 | 1 |  |  |  |  | 1 |  | 1 |  |  | 1 |  |  | 1 |  | 1 |
| 15 | Applies half drape |  |  |  |  |  |  |  |  |  |  | 1 |  |  |  |  |  |  |  |  |  |  |  |  |  |  |
| 16 | Drape, size not specified | 1 |  |  |  |  | 1 |  |  |  |  |  |  |  | 1 |  |  |  | 0.5 |  |  |  | 0.33 |  |  |  |
| 17 | Indication noted |  |  |  |  |  |  |  |  |  |  |  |  |  |  |  |  |  |  |  | 1 |  |  |  |  |  |
| 18 | Identifies anatomic landmarks (site selection) | 1 |  |  |  |  | 1 |  |  |  |  | 1 | 2 |  |  |  | 1 |  | 1 |  |  |  | 1 | 1 | 1 |  |
| 19 | Identifies carotid by palpation |  |  |  |  |  |  |  |  |  |  |  |  |  |  |  |  |  |  |  |  |  | 1 |  |  |  |
| 20 | Turns on machine | 1 |  |  |  |  |  |  |  |  |  |  |  |  |  |  |  |  |  |  |  |  |  |  |  |  |
| 21 | Places gel on probe | 1 |  |  |  |  |  |  |  |  |  |  |  |  |  |  |  |  |  |  |  |  |  |  |  |  |
| 22 | Appropriate gain | 1 |  |  |  |  |  |  |  |  |  |  |  |  |  |  |  |  |  |  |  |  |  |  |  |  |
| 23 | Appropriate orientation (indicator to left) | 1 |  |  |  |  |  |  |  |  |  |  |  |  |  |  |  |  |  |  |  |  |  |  |  |  |
| 24 | US to identify IJ | 0.5 |  |  |  |  |  |  |  |  |  |  |  |  |  |  |  |  |  |  |  |  |  | 0.5 |  |  |
| 25 | US to identify carotid artery | 0.5 |  |  |  |  |  |  |  |  |  |  |  |  |  |  |  |  |  |  |  |  |  | 0.5 |  |  |
| 26 | Specifies Transverse vs Longitudinal | 1 |  |  |  |  |  |  |  |  |  |  |  |  |  |  |  |  |  |  |  |  |  |  |  |  |
| 27 | IJ compressibility by ultrasound (after sterilization) |  |  |  |  |  |  |  |  |  | 1 |  |  |  |  |  |  |  |  |  |  |  |  |  |  |  |
| 28 | Lidocaine into syringe | 1 |  |  |  |  |  |  |  |  |  |  |  |  |  |  |  |  |  |  |  |  |  | 1 |  |  |
| 29 | Prepares cannulation needle (accessible or attach to syringe) | 1 |  |  |  |  |  |  |  |  |  | 0.25 |  |  |  |  |  |  |  |  |  |  |  |  |  |  |
| 30 | Aligns bevel |  |  |  |  |  |  |  |  |  |  | 0.25 |  |  |  |  |  |  |  |  |  |  |  |  |  |  |
| 31 | Dilator easily accessible | 1 |  |  |  |  |  |  |  |  |  |  |  |  |  |  |  |  |  |  |  |  |  |  |  |  |
| 32 | Flushes ports |  | 1 | 1 |  |  |  |  |  |  |  |  |  |  |  |  |  |  |  |  |  |  |  |  |  |  |
| 33 | Clamps ports |  | 1 | 1 |  |  |  |  |  |  |  | 0.25 |  |  |  |  |  |  |  |  |  |  |  |  |  |  |
| 34 | Removes brown port cap | 1 | 1 |  |  |  |  |  |  |  |  | 0.25 |  |  |  |  |  |  |  |  |  |  | 1 |  |  |  |
| 35 | Prepares catheter, not otherwise specified |  |  |  |  |  |  |  |  |  |  |  |  |  |  |  |  |  |  |  |  |  |  | 1 |  |  |
| 36 | Prepares guide wire, place on drape | 1 |  |  |  |  |  |  |  |  |  |  |  |  |  |  |  |  |  |  |  |  |  |  |  |  |
| 37 | Prepares scalpel and suture | 1 |  |  |  |  |  |  |  |  |  |  |  |  |  |  |  |  |  |  |  |  |  |  |  |  |
| 38 | Establishes distance of (prepare) catheter |  |  |  |  |  |  |  |  |  |  |  |  | 1 |  |  |  |  |  |  |  |  |  |  |  |  |
| 39 | Knowledge of instruments |  |  |  |  |  |  |  |  |  |  | 1 |  |  |  |  | 1 |  |  |  |  |  | 1 | 1 |  |  |
| 40 | Inappropriate preparation |  |  |  |  |  | 1 |  |  |  |  |  |  |  |  |  | 1 |  |  |  |  |  | 0.33 |  | 1 |  |
| 41 | Inappropriate setup |  |  |  |  |  | 1 |  |  |  |  |  |  |  |  |  |  |  |  |  |  |  |  |  |  |  |
| 42 | Places ultrasound gel into cover, sleeve | 1 |  |  |  |  |  |  |  |  |  |  |  | 1 |  |  |  |  |  |  |  |  |  |  |  |  |
| 43 | Inserts vascular probe into sterile sheath | 1 | 0.5 | 0.5 |  |  |  |  |  |  |  |  |  | 1 |  |  |  |  |  |  |  |  |  | 1 |  |  |
| 44 | Places rubberband 1 at tip of probe | 1 |  |  |  |  |  |  |  |  |  |  |  |  |  |  |  |  |  |  |  |  |  |  |  |  |
| 45 | Places rubberband 2 at distal cord | 1 |  |  |  |  |  |  |  |  |  |  |  |  |  |  |  |  |  |  |  |  |  |  |  |  |
| 46 | Places sterile probe onto field | 1 |  |  |  |  |  |  |  |  |  |  |  |  |  |  |  |  |  |  |  |  |  |  |  |  |
| 47 | Places sterile gel onto skin | 1 | 0.5 | 0.5 |  |  |  |  |  |  |  |  |  | 1 |  |  |  |  |  |  |  |  |  |  |  |  |
| 48 | Locates carotid artery and IJ with US | 1 | 1 | 1 |  |  |  |  |  |  |  |  |  |  |  |  |  |  |  |  |  |  |  | 1 |  |  |
| 49 | General Sterile ultrasound technique |  |  |  |  |  |  |  |  |  | 1 |  |  |  |  |  |  |  |  |  |  |  |  |  |  |  |
| 50 | Hands off pager |  |  |  |  |  |  |  |  |  |  |  |  |  |  |  |  |  |  |  | 1 |  |  |  |  |  |
| 51 | Washes hands |  |  |  | 1 |  | 1 |  | 1 |  | 1.2 | 1 |  |  | 0.5 | 1 |  | 1 |  |  | 1 | 1 |  | 1 |  |  |
| 52 | Max sterile barrier, not otherwise specified |  |  |  |  |  |  |  |  |  |  |  |  |  |  |  |  |  |  |  |  | 1 |  |  |  |  |
| 53 | Standard precautions - face shield/mask | 1 | 0.25 | 0.33 | 0.33 |  |  |  | 1 | 2 | 0.2 | 0.25 |  |  | 1 | 0.2 |  | 0.25 |  |  | 1 |  |  | 0.25 |  | 1 |
| 54 | Standard precautions - hat | 1 | 0.25 |  | 0.33 |  |  |  |  | 1 | 0.2 | 0.25 |  |  | 1 | 0.2 |  | 0.25 |  |  |  |  |  | 0.25 |  | 1 |
| 55 | Standard precautions - gown | 1 | 0.25 | 0.33 | 0.33 |  |  |  | 1 | 1 | 0.7 | 0.25 |  |  | 1 | 0.2 |  | 0.25 |  |  | 1 |  |  | 0.25 |  | 1 |
| 56 | Gown tied |  |  |  |  |  |  |  |  |  |  |  |  |  |  |  |  |  |  |  |  |  |  |  |  | 1 |
| 57 | Standard precautions - sterile gloves | 1 | 0.25 | 0.33 | 1 |  |  |  | 1 | 1 | 0.7 | 0.25 |  | 1 | 1 | 0.2 |  | 0.25 |  |  | 1 |  |  | 0.25 |  | 1 |
| 58 | Injects lidocaine | 1 | 1 | 1 |  |  |  |  |  |  |  | 1 |  |  |  |  |  |  | 1 |  |  |  |  | 1 |  |  |
| 59 | Anesthetizes deeper structures |  | 1 | 1 |  |  |  |  |  |  |  |  |  |  |  |  |  |  |  |  |  |  |  |  |  |  |
| 60 | Localizes vein with anesthetizing needle |  |  |  |  |  |  |  |  |  |  |  |  |  |  |  |  |  |  |  |  |  |  |  |  |  |
| 61 | Chooses the correct needle |  |  |  |  |  |  |  |  |  |  | 1 |  |  |  |  |  |  |  |  |  |  |  |  |  |  |
| 62 | Cannulates vein with finder needle (attempts) | 1 |  |  |  |  |  |  |  |  |  |  |  |  |  |  |  |  |  |  |  |  |  |  |  |  |
| 63 | Needle insertion at proper angle, trajectory |  |  |  |  |  | 1 |  |  |  |  | 4 | 1 |  |  |  | 1 |  |  |  |  |  | 1 |  | 1 |  |
| 64 | Needle too cephalad |  |  |  |  |  |  |  |  |  |  |  | 1 |  |  |  |  |  |  |  |  |  |  |  |  |  |
| 65 | Aspirates while advancing |  | 0.5 | 0.5 |  |  |  |  |  |  |  | 1 |  |  |  |  | 1 |  |  |  |  |  |  |  | 1 |  |
| 66 | Cannulates vein with large bore needle (attempts) | 1 |  |  |  |  |  |  |  |  |  | 1 |  |  |  |  |  |  | 1 |  |  |  |  |  |  |  |
| 67 | Redirects needle if unsuccessful |  |  |  |  |  |  |  |  |  |  | 1 |  |  |  |  |  |  |  |  |  |  |  |  |  |  |
| 68 | Cannulates vein (specify with ultrasound) |  | 0.5 | 0.5 |  |  |  |  |  |  |  |  |  |  |  |  |  |  |  |  |  |  |  | 1 |  |  |
| 69 | Advances needle |  |  |  |  |  |  |  |  |  |  |  |  |  |  |  |  |  |  |  |  |  | 1 |  |  |  |
| 70 | Removes syringe |  | 1 | 1 |  |  |  |  |  |  |  |  |  |  |  |  |  |  |  |  |  |  | 1 |  |  |  |
| 71 | Removes syringe, specifies without moving needle |  |  |  |  |  |  |  |  |  |  | 1 | 1 |  |  |  |  |  |  |  |  |  |  |  |  |  |
| 72 | Venous blood in syringe, confirm puncture | 1 |  |  |  |  |  |  |  |  |  |  |  | 1 |  |  |  |  |  |  |  |  | 1 |  |  |  |
| 73 | Threads guide wire through needle (attempts) | 1 |  |  |  |  |  |  |  |  |  | 1 |  | 1 |  |  | 1 |  |  |  |  |  |  |  | 1 |  |
| 74 | Advances wire (distance not specified) |  |  |  |  |  |  |  |  |  |  |  |  | 1 |  |  |  |  |  |  |  |  |  |  |  |  |
| 75 | Advances wire no more than 12-15 cm |  | 1 | 1 |  |  |  |  |  |  |  |  |  |  |  |  |  |  |  |  |  |  |  |  |  |  |
| 76 | Advances wire no more than 20cm |  |  |  |  |  |  |  |  |  |  |  |  | 1 |  |  |  |  |  |  |  |  | 1 |  |  |  |
| 77 | Extends skin incision with scalpel | 1 | 1 | 1 |  |  |  |  |  |  |  |  |  |  |  |  | 0.5 |  |  |  |  |  | 1 | 0.5 | 0.5 |  |
| 78 | Removes needle | 1 |  |  |  |  |  |  |  |  |  |  |  | 0.5 |  |  | 0.5 |  |  |  |  |  | 1 |  | 0.5 |  |
| 79 | Dilates tract with dilator | 1 | 1 | 1 |  |  |  |  |  |  |  |  |  |  |  |  |  |  |  |  |  |  | 1 | 0.5 |  |  |
| 80 | Withdraws dilator |  |  |  |  |  |  |  |  |  |  |  |  |  |  |  |  |  |  |  |  |  | 1 |  |  |  |
| 81 | CV catheter over guide wire | 0.5 | 1 | 1 |  |  | 1 |  |  |  |  |  |  | 0.5 |  |  | 0.5 |  |  |  |  |  |  | 0.5 | 0.5 |  |
| 82 | Hand on wire all times | 0.5 | 1 | 1 |  |  |  |  |  |  |  |  |  | 1 |  |  |  |  |  |  |  |  | 1 |  |  |  |
| 83 | Removes guide wire | 1 | 1 | 1 |  |  |  |  |  |  |  |  |  | 1 |  |  | 0.5 |  |  |  |  |  | 1 | 0.5 | 0.5 |  |
| 84 | Guidewire technique, not otherwise specified |  |  |  |  |  |  |  |  |  |  |  |  |  |  |  |  |  | 1 |  |  |  |  | 1 |  |  |
| 85 | Advances catheter (14-16 cm) |  | 1 | 1 |  |  |  |  |  |  |  |  |  |  |  |  |  |  |  |  |  |  | 1 |  |  |  |
| 86 | Advances catheter (distance not specified) |  |  |  |  |  |  |  |  |  |  |  |  | 1 |  |  |  |  |  |  |  |  |  |  |  |  |
| 87 | Procedural pause |  |  |  |  |  |  |  |  | 1 | 1 | 1 |  |  |  |  |  |  |  |  |  |  |  |  |  |  |
| 88 | Obtains blood return (in all three ports) | 1 | 0.5 | 0.5 |  |  |  |  |  |  |  |  |  | 0.5 |  |  | 0.5 |  |  |  |  |  | 1 | 0.5 | 0.5 |  |
| 89 | Flushes all ports with saline | 1 | 0.5 | 0.5 |  |  |  |  |  |  |  |  |  | 0.5 |  |  | 0.5 |  |  |  |  |  | 0.5 | 0.5 | 0.5 |  |
| 90 | Heparin flush |  |  | 1 |  |  |  |  |  |  |  |  |  |  |  |  |  |  |  |  |  |  |  |  |  |  |
| 91 | Clamps each port |  |  |  |  |  |  |  |  |  |  |  |  |  |  |  | 1 |  |  |  |  |  | 0.5 |  | 1 |  |
| 92 | Places clip at skin site | 1 |  |  |  |  |  |  |  |  |  |  |  |  |  |  |  |  |  |  |  |  | 1 |  |  |  |
| 93 | Sutures CVC (in four sites) | 1 | 1 | 1 |  |  |  |  | 1 |  | 1 |  |  |  |  |  | 1 |  |  |  |  |  | 1 |  | 1 |  |
| 94 | Applies sterile dressing | 1 |  | 1 | 1 |  |  |  | 1 | 1 |  |  |  |  |  | 1 |  | 1 |  |  |  |  |  |  | 1 |  |
| 95 | Applies dressing (sterility not specified) |  | 1 |  |  |  |  |  |  |  |  |  |  |  |  |  |  |  |  |  | 1 |  |  |  |  |  |
| 96 | Dressing type noted |  |  |  |  |  |  |  |  |  |  |  |  |  |  |  |  |  |  |  | 1 |  |  |  |  |  |
| 97 | Avoids antibiotic ointment |  |  |  |  |  |  |  | 1 |  |  |  |  |  |  |  |  |  |  |  |  |  |  |  |  |  |
| 98 | Hand hygiene post-procedure |  |  |  |  |  |  |  |  |  |  |  |  |  | 1 |  |  |  |  |  |  |  |  |  |  |  |
| 99 | US to confirm venous access |  |  |  |  | 1 |  |  |  |  | 1 |  |  |  |  |  |  |  |  | 1 |  |  |  |  |  |  |
| 100 | Knowledge of procedure |  |  |  |  |  |  |  |  |  |  |  |  |  |  |  |  |  |  |  |  |  | 1 |  |  |  |
| 101 | Removes all sharps from site | 1 |  |  |  |  |  |  |  |  |  |  |  |  |  |  |  |  |  |  |  |  |  | 1 |  |  |
| 102 | Removes biohazards |  |  |  |  |  |  |  |  |  |  |  |  |  |  |  |  |  |  |  |  |  |  | 1 |  |  |
| 103 | CXR | 1 | 1 | 1 |  |  |  |  |  |  |  |  |  |  |  |  |  |  |  |  | 1 |  | 1 | 1 |  |  |
| 104 | Documents procedure | 1 |  |  |  |  |  |  |  |  |  |  |  |  |  |  |  |  |  |  |  |  |  |  |  |  |
| 105 | Notify catheter ok to use |  | 1 | 1 |  |  |  |  |  |  |  |  |  |  |  |  |  |  |  |  |  |  |  |  |  |  |
| 106 | Documents complications | 1 |  |  |  |  |  |  |  |  |  |  |  |  |  |  |  |  |  |  |  |  |  |  |  |  |
| 107 | Type justified |  |  |  |  |  |  |  |  |  |  |  |  |  |  |  |  |  |  |  |  |  |  |  | 1 |  |
| 108 | Type observed and recorded | -- |  |  |  |  |  |  |  |  |  |  |  |  |  |  |  |  |  |  | 1 |  |  |  |  |  |
| 109 | Choice of site noted | -- |  |  |  |  |  |  |  |  | 1 |  |  |  |  |  |  |  |  |  | 1 |  |  |  | 1 | 1 |
| 110 | Specifies choosing SC site over others |  |  |  |  |  |  |  | 1 |  |  | 1 |  |  |  |  |  |  |  |  |  |  |  |  |  |  |
| 111 | Approach noted (eg - supraclavicular/infraclavicular) | -- |  |  |  |  |  |  |  |  |  |  |  |  |  |  |  |  |  |  |  |  |  |  |  |  |
| 112 | Side observed and recorded | -- |  |  |  |  |  |  |  |  |  |  |  |  |  |  |  |  |  |  | 1 |  |  |  |  |  |
| 113 | Landmark technique only | -- |  |  |  |  |  |  |  |  |  |  |  |  |  |  |  |  |  |  |  |  |  |  |  |  |
| 114 | Ultrasound localization landmark | -- |  |  |  |  |  |  |  |  |  |  |  |  |  |  |  |  |  |  |  |  |  |  |  |  |
| 115 | Real-time ultrasound guided / bimanual use of US and needle | -- |  |  |  |  |  |  |  |  |  |  |  |  |  |  |  |  |  |  |  |  |  | 1 |  |  |
| 116 | Landmark failure U/S guided | -- |  |  |  |  |  |  |  |  |  |  |  |  |  |  |  |  |  |  |  |  |  |  |  |  |
| 117 | Respect for Tissue |  |  |  |  |  |  |  |  |  |  |  |  |  |  |  |  |  |  |  |  |  | 1 |  |  |  |
| 118 | Economy and smoothness of movements |  |  |  |  |  |  |  |  |  |  |  |  |  |  |  | 1 |  |  |  |  |  | 1 |  |  |  |
| 119 | Instrument handling |  |  |  |  |  |  |  |  |  |  |  |  |  |  |  | 1 |  |  |  |  |  | 1 |  |  |  |
| 120 | Use of assistants |  |  |  |  |  |  |  |  | 1 |  |  |  |  |  |  |  |  |  |  |  |  | 1 |  |  |  |
| 121 | Flow |  |  |  |  |  |  |  |  |  |  |  |  |  |  |  | 1 |  |  |  |  |  | 1 |  |  |  |
| 122 | Overall performance/ability |  |  |  |  |  | 1 |  |  |  |  | 1 |  | 1 |  |  | 1 |  | 1 |  |  |  |  |  |  |  |
| 123 | Overall comfort |  |  |  |  |  | 1 |  |  |  |  |  |  |  |  |  |  |  |  |  |  |  |  |  |  |  |
| 124 | Total number of attempts insert |  |  |  |  |  |  | 1 |  |  |  |  | 1 | 1 |  |  | 1 |  |  |  | 1 |  |  |  | 1 |  |
| 125 | Total number of attempts locate | 1 |  |  |  |  | 1 |  |  |  | 1 | 1 | 1 |  |  |  | 1 |  |  |  | 1 |  |  |  | 1 |  |
| 126 | Successful CVC insertion by operator | 1 |  |  |  |  | 1 |  |  |  | 1 |  |  | 1 |  |  |  |  | 1 |  |  |  |  |  |  |  |
| 127 | Successful CVC insertion, specify on first attempt |  |  |  |  |  | 1 |  |  |  |  |  |  |  |  |  |  |  |  |  |  |  |  |  |  |  |
| 128 | Specifies independent |  |  |  |  |  |  |  |  |  | 1 |  |  |  |  |  |  |  |  |  |  |  |  |  |  |  |
| 129 | Help needed (any help) | 1 |  |  |  |  | 1 |  |  |  |  |  |  |  |  |  |  |  |  |  |  |  |  |  | 1 |  |
| 130 | Proper supervision |  |  |  |  |  |  |  |  | 2 |  |  |  |  |  |  |  |  |  |  | 1 |  |  |  |  |  |
| 131 | Time to completion | 1 |  |  |  |  |  | 1 |  |  | 1 |  |  | 1 |  |  | 1 |  |  | 1 |  |  |  |  | 1 |  |
| 132 | Pneumothorax | 1 |  |  |  |  | 1 |  |  |  |  |  |  |  |  |  |  |  |  |  | 1 |  |  |  |  |  |
| 133 | Hemothorax | 1 |  |  |  |  |  |  |  |  |  |  |  |  |  |  |  |  |  |  |  |  |  |  |  |  |
| 134 | Hemomediastinum | 1 |  |  |  |  |  |  |  |  |  |  |  |  |  |  |  |  |  |  |  |  |  |  |  |  |
| 135 | Vessel laceration | 1 |  |  |  |  |  |  |  |  |  |  |  |  |  |  |  |  |  |  |  |  |  |  |  |  |
| 136 | Malignant dysrhythmia | 1 |  |  |  |  |  |  |  |  |  |  |  |  |  |  |  |  |  |  |  |  |  |  |  |  |
| 137 | Air embolus | 1 |  |  |  |  |  |  |  |  |  |  |  |  |  |  |  |  |  |  |  |  |  |  |  |  |
| 138 | Hematoma | 1 |  |  |  |  |  |  |  |  |  |  |  |  |  |  |  |  |  |  |  |  |  |  |  |  |
| 139 | Transient catheter malposition | 1 |  |  |  |  | 1 |  |  |  |  |  |  |  |  |  |  |  |  |  | 1 |  |  |  |  |  |
| 140 | Arterial puncture with or without significant hemorrhage | 1 |  |  |  | 1 | 1 | 1 |  |  |  |  |  |  |  |  |  |  |  |  |  |  |  |  |  |  |
| 141 | Break in sterile technique/maintain sterile technique (number of times) | 1 | 1 | 1 | 1 |  |  |  |  |  |  | 1 |  |  |  |  |  | 1 |  |  | 1 |  |  |  |  | 3 |
| 142 | Through periosteum |  |  |  |  |  |  |  |  |  |  |  | 1 |  |  |  |  |  |  |  |  |  |  |  |  |  |
| 143 | Did all other personnel follow sterile procedures? |  |  |  | 1 |  |  |  |  | 1 |  |  |  |  |  | 0.2 |  | 1 |  |  |  |  |  |  |  |  |
| 144 | Number of times posterior wall penetrated |  |  |  |  | 1 |  |  |  |  |  |  |  |  |  |  |  |  |  |  |  |  |  |  |  |  |
| 145 | Responds to monitor with SVT/VT |  |  |  |  |  |  |  |  |  |  |  |  |  |  |  |  |  |  |  |  |  |  |  |  |  |
| 146 | Emergent/elective |  |  |  |  |  |  |  |  |  |  |  |  |  |  |  |  |  |  |  | 1 |  |  |  |  |  |
| 147 | Mayo stand cover |  |  |  |  |  |  |  |  |  |  |  |  |  |  |  |  |  |  |  |  |  |  | 1 |  |  |
